# Reversing methanogenesis to capture methane for liquid biofuel precursors

**DOI:** 10.1186/s12934-015-0397-z

**Published:** 2016-01-14

**Authors:** Valerie W. C. Soo, Michael J. McAnulty, Arti Tripathi, Fayin Zhu, Limin Zhang, Emmanuel Hatzakis, Philip B. Smith, Saumya Agrawal, Hadi Nazem-Bokaee, Saratram Gopalakrishnan, Howard M. Salis, James G. Ferry, Costas D. Maranas, Andrew D. Patterson, Thomas K. Wood

**Affiliations:** Department of Chemical Engineering, The Pennsylvania State University, University Park, PA 16802-4400 USA; Department of Veterinary and Biomedical Sciences, The Pennsylvania State University, University Park, PA 16802-4400 USA; Department of Chemistry, The Pennsylvania State University, University Park, PA 16802-4400 USA; Department of Biochemistry and Molecular Biology, The Pennsylvania State University, University Park, PA 16802-4400 USA; The Huck Institutes of the Life Sciences, The Pennsylvania State University, University Park, PA 16802-4400 USA; Key Laboratory of Magnetic Resonance in Biological Systems, Wuhan Institute of Physics and Mathematics, Chinese Academy of Sciences, Wuhan, 430071 China; Institute of Natural and Mathematical Sciences, Massey University, Auckland, 0632 New Zealand

**Keywords:** Reverse methanogenesis, Anaerobic oxidation of methane, Methyl-coenzyme M reductase

## Abstract

**Background:**

Energy from remote methane reserves is transformative; however, unintended release of this potent greenhouse gas makes it imperative to convert methane efficiently into more readily transported biofuels. No pure microbial culture that grows on methane anaerobically has been isolated, despite that methane capture through anaerobic processes is more efficient than aerobic ones.

**Results:**

Here we engineered the archaeal methanogen *Methanosarcina acetivorans* to grow anaerobically on methane as a pure culture and to convert methane into the biofuel precursor acetate. To capture methane, we cloned the enzyme methyl-coenzyme M reductase (Mcr) from an unculturable organism, anaerobic methanotrophic archaeal population 1 (ANME-1) from a Black Sea mat, into *M. acetivorans* to effectively run methanogenesis in reverse. Starting with low-density inocula, *M. acetivorans* cells producing ANME-1 Mcr consumed up to 9 ± 1 % of methane (corresponding to 109 ± 12 µmol of methane) after 6 weeks of anaerobic growth on methane and utilized 10 mM FeCl_3_ as an electron acceptor. Accordingly, increases in cell density and total protein were observed as cells grew on methane in a biofilm on solid FeCl_3_. When incubated on methane for 5 days, high-densities of ANME-1 Mcr-producing *M. acetivorans* cells consumed 15 ± 2 % methane (corresponding to 143 ± 16 µmol of methane), and produced 10.3 ± 0.8 mM acetate (corresponding to 52 ± 4 µmol of acetate). We further confirmed the growth on methane and acetate production using ^13^C isotopic labeling of methane and bicarbonate coupled with nuclear magnetic resonance and gas chromatography/mass spectroscopy, as well as RNA sequencing.

**Conclusions:**

We anticipate that our metabolically-engineered strain will provide insights into how methane is cycled in the environment by Archaea as well as will possibly be utilized to convert remote sources of methane into more easily transported biofuels via acetate.

**Electronic supplementary material:**

The online version of this article (doi:10.1186/s12934-015-0397-z) contains supplementary material, which is available to authorized users.

## Background

Advances in horizontal drilling since the late 1970s have made methane resources economical through hydraulic fracturing [1]. All continents, except Antarctica, are known to possess recoverable shale reserves, with a global amount of shale gas totaling up to 7300 trillion cubic feet [2]. The major component of shale gas is methane, a potent greenhouse gas that is 25 times more damaging than carbon dioxide (CO_2_) over a span of 100 years [3]. To exploit the abundance of these energy sources and to reduce methane emissions, state-of-the-art chemical plants employ Fischer–Tropsch processes to convert methane into liquid fuels (e.g., Shell Pearl Facility in Qatar with an output of 260,000 barrels of oil per day [4]); however, these plants rely on complex technology that demands large-scale investment [5]. An alternative approach is to biologically convert methane to liquid fuels, a method that may be more suitable for the thousands of remote fracking sites. Biological conversion of methane is a more economically and environmentally sustainable technology, since it requires a smaller footprint and is less technologically complex [6]. Hence, we explored methane activation using an anaerobe as our biological catalyst and focused on anaerobic processes, as they confer higher energy and carbon yield efficiencies with lower CO_2_ emissions than aerobic ones for converting methane into fuels [6]. Furthermore, creating biofuels from methane should have a high impact for second and third generation biofuels since they will compete on yield and feedstock cost rather than on fermentation time.

Anaerobic oxidation of methane (AOM) is a key regulator of global fluxes of methane and the carbon cycle, capturing up to 300 Tg of methane per year [7]. However, AOM is a little-understood biological process driven by natural consortia consisting of ANME and syntrophic bacteria. Despite decades of effort, these natural consortia have never been successfully isolated, most probably due to their long lag phase (~60 years) [8] and doubling time (~7 months) [9]. Further, separate cultivation of ANME and their syntrophic bacteria has not been reported.

The first step to anaerobically activate methane in a process known as reverse methanogenesis is likely to be catalyzed by Mcr, as suggested by the prevalence of *mcr* genes in ANME populations [10]. Trace AOM has also been observed in anaerobic methanogens such as *Methanothermobacter marburgensis* [11] and *M. acetivorans* [12, 13]; however, none of these strains utilize methane as the major energy and carbon source for growth.

Here we engineered an anaerobic archaeal strain that uses methane and bicarbonate as the major energy and carbon sources to support its growth. Our ultimate goal is to produce liquid fuels efficiently via anaerobic processes, and for this reason, we set out to create a system that could capture methane using simple technology on a laboratory scale. We cloned the genes encoding Mcr from the metagenome of an unculturable ANME-1 population originally identified in Black Sea mats [14, 15], where AOM rates are the highest in all studied aquatic environments [7]. *M. acetivorans* was chosen as the host, as this strain is metabolically diverse since it has the largest archaeal genome [16], is genetically tractable [17], and encodes a native Mcr for producing methane during methanogenesis [CH_3_-SCoM + HSCoB **→** CH_4_ + CoBS-SCoM, where CH_3_-SCoM is methyl-coenzyme M (2-(methylthio)ethanesulfonate), HSCoB is coenzyme B (7-mercaptoheptanoylthreonine phosphate), and CoBS-SCoM is the heterodisulfide of coenzyme B and coenzyme M]. Therefore, we reasoned this host has the necessary cofactors and cellular machinery to produce active ANME-1 Mcr.

## Results and discussion

### Growth on methane via ANME-1 Mcr

In contrast to previous studies where trace AOM was detected in *M. acetivorans* [12, 13], we forced the engineered cells, *M. acetivorans* with cloned ANME-1 *mcr* genes from the Black Sea metagenome, to utilize methane for growth by not providing an additional carbon source (e.g., methanol, acetate, trimethylamine). Instead, we added various electron acceptors (Fe^3+^, NO_3_^−^, NO_2_^−^, SO_4_^2−^, and Mn^4+^) that could remove the electrons generated by growth on methane and to mimic the syntrophic role of sulfate-reducing bacteria associated with ANME-1 organisms. We then characterized the ability of the engineered strain to catabolize methane using growth-based measurements, ^13^C isotopic labeling of methane and bicarbonate, and RNA sequencing.

**Fig. 1:**
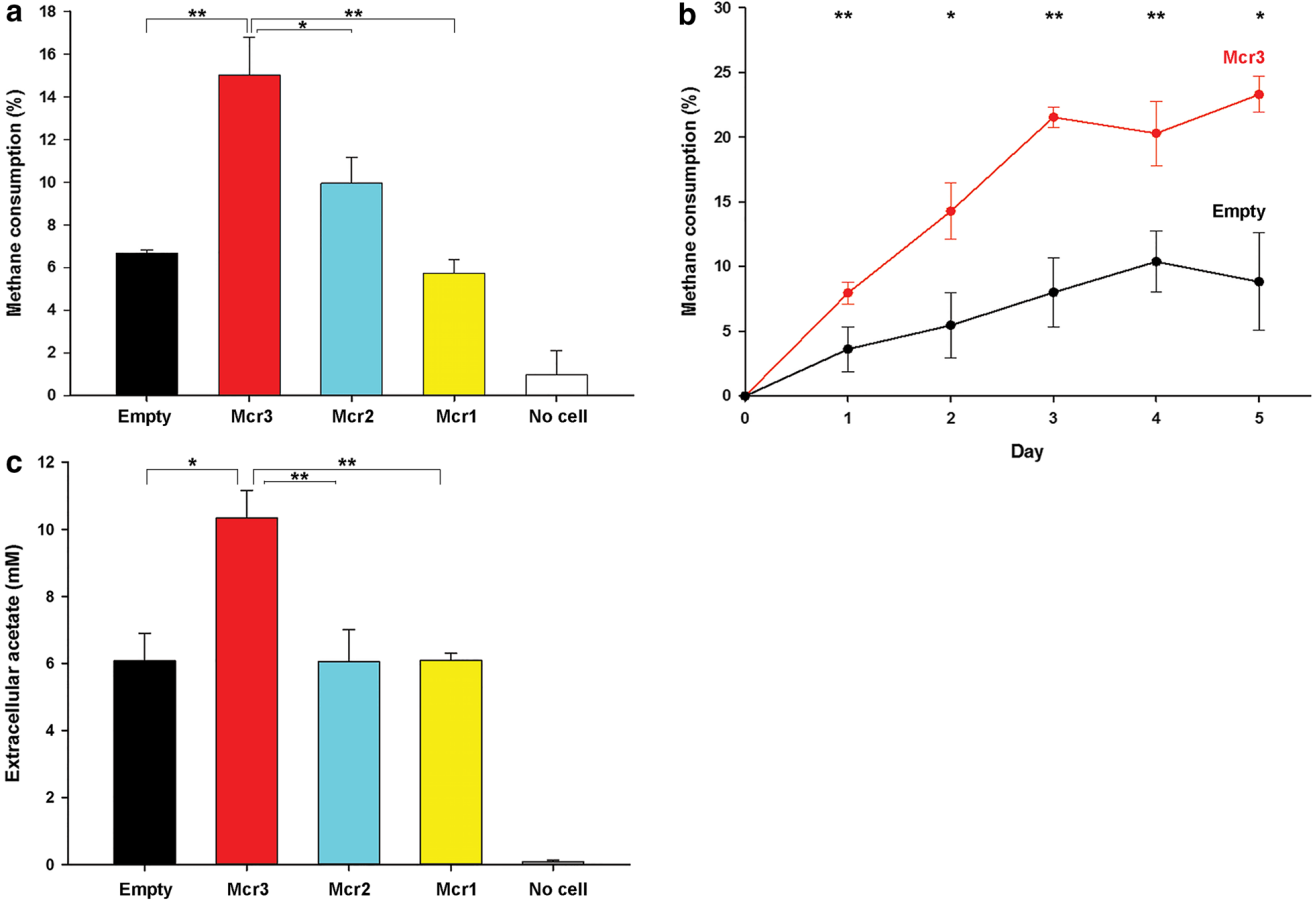
Methane consumption by ANME-1 Mcr-producing *M. acetivorans* during high-density growth experiments. All values are presented as mean ± sem of three cultures. **P ≤ 0.05, *P < 0.15. **a** Methane consumption of cells harboring pES1-MAT*mcr*1 (*Mcr1*), pES1-MAT*mcr*2 (*Mcr2*), pES1-MAT*mcr*3 (*Mcr3*), and pES1(Pmat) (*Empty*) after 5 days showing that pES1-MAT*mcr*3 is the best construct in performing reverse methanogenesis in *M. acetivorans*. The absence of cells (*No cells*) in the same growth medium (HS-methane medium and 0.1 mM FeCl_3_ and puromycin) showing negligible methane reduction confirmed minimal gas leakage from the tubes. **b** Increase in methane consumption by high cell-density cultures of *M. acetivorans*/pES1-MAT*mcr*3 (*Mcr3*) in comparison to pES1(Pmat)-harboring cells (*Empty*) for 5 days showing the importance of the cloned *mcr*. **c** Higher production of extracellular acetate was observed for high cell-density cultures of *M. acetivorans*/pES1-MAT*mcr*3 (*Mcr3*) than for *M. acetivorans*/pES1(Pmat) (*Empty*), *M. acetivorans*/pES1-MAT*mcr*2 (*Mcr2*), and *M. acetivorans*/pES1-MAT*mcr*1 (*Mcr1*) after 5 days on methane and 0.1 mM FeCl_3_. The absence of cells (*No cells*) in the same growth medium (HS-methane medium with 0.1 mM FeCl_3_ and puromycin) showing negligible extracellular acetate confirmed minimal abiotic formation of acetate

The ANME-1 *mcrBGA* genes encoding for ANME-1 Mcr were cloned into *M. acetivorans* using vector pES1 [18] to form pES1-MAT*mcr*3 which expresses *mcrBGA* using its native promoter P_mcr_ANME-1_. This native promoter was found to be superior to that of the CO dehydrogenase/acetyl-CoA synthase promoter (P_cdh_) from *Methanosarcina thermophila* [18] and the *mcr* promoter from *M. acetivorans* (P_mcr_*M. acetivorans*_) (Additional file 1: Figure S1). To compare the promoters, we conducted short-duration (5 days) experiments that utilized high densities of cells grown first on methanol (10^9^ CFU/mL), which were subsequently added to minimal high-salt (HS) medium (Additional file 1: Table S1) with methane and bicarbonate and tested for the amount of methane utilization. In cells exposed to methane, ANME-1 Mcr was produced in *M. acetivorans* by P_cdh_ and P_mcr_ANME-1_, as shown by the appearance of ANME-1 McrA-FLAG (the α subunit of the ANME-1 Mcr complex that was tagged with a FLAG epitope) in a Western blot (Additional file 1: Figure S2). Corroborating the production of ANME-1 Mcr by P_mcr_ANME-1_, methane consumption using whole cells was the highest when P_mcr_ANME-1_ was used to express ANME-1 *mcr* (15 ± 1.7 %, corresponding to 143 ± 16 µmol of methane, Fig. 1a). Significant differences between ANME-1 Mcr-producing cultures and those harboring an empty plasmid were observed throughout 5 days (Fig. 1b); therefore, the native ANME-1 *mcr* promoter was used for the remainder of the experiments. Note that absolute amounts of reacting species are indicated in µmol to provide a more direct comparison between liquid products and gaseous substrates since the volume of the headspace and the volume of the liquid in each culture tube of different experiments were not the same.

**Fig. 2:**
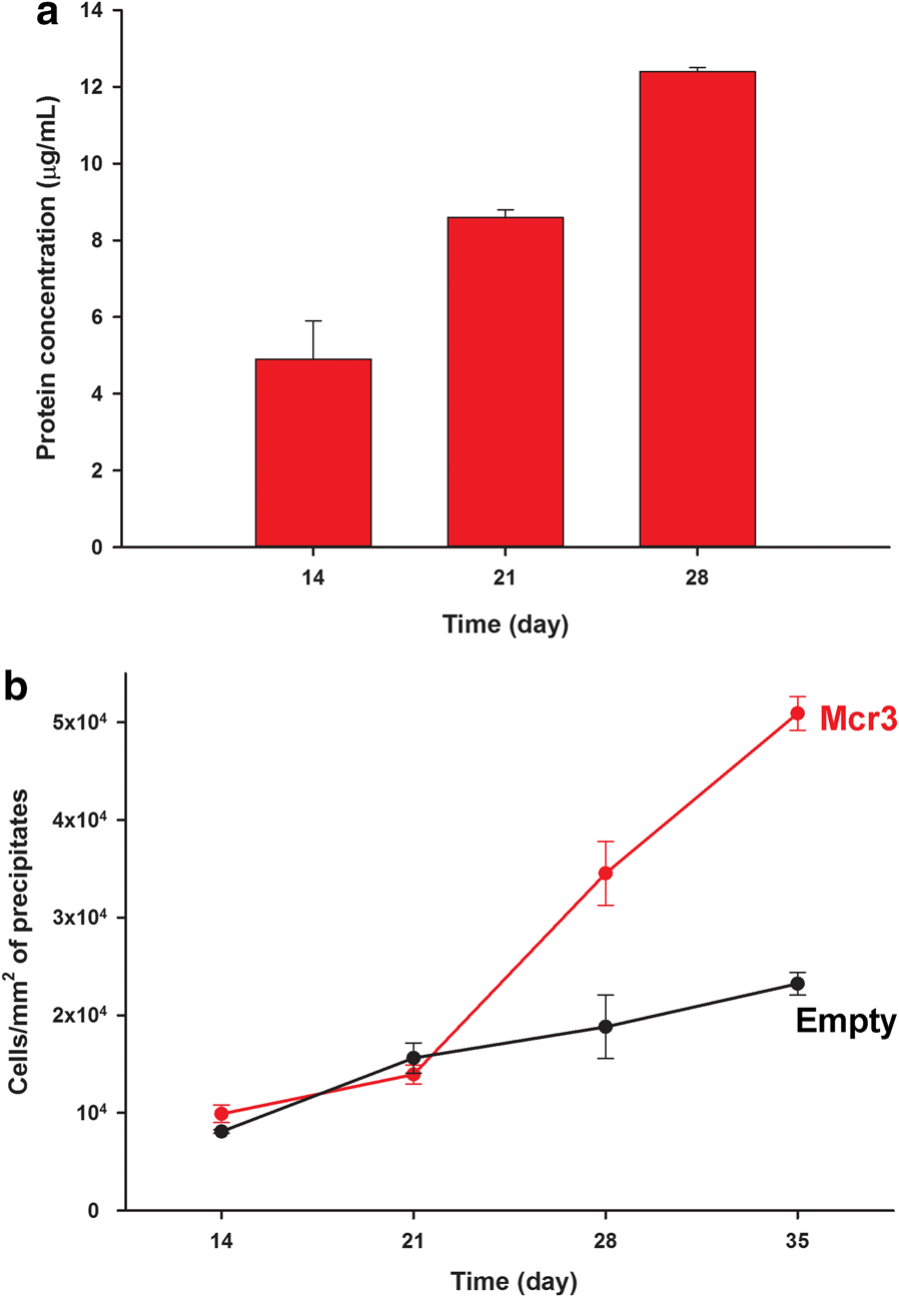
Long-term growth of ANME-1 Mcr-producing *M. acetivorans* on methane and 10 mM FeCl_3_ during low-density growth experiments. **a** Total protein concentration of a representative culture of *M. acetivorans*/pES1-MAT*mcr*3 grown on methane and 10 mM FeCl_3_ after 14, 21, and 28 days of incubation. Values are presented as means ± standard deviations. **b** Number of *M. acetivorans*/pES1-MAT*mcr*3 (*Mcr3*) and *M. acetivorans*/pES1(Pmat) (*Empty*) cells per area (mm^2^) of precipitates after 14, 21, 28, and 35 days of incubation on methane and 10 mM FeCl_3_. Values are presented as mean ± sem of three or more cultures

**Fig. 3:**
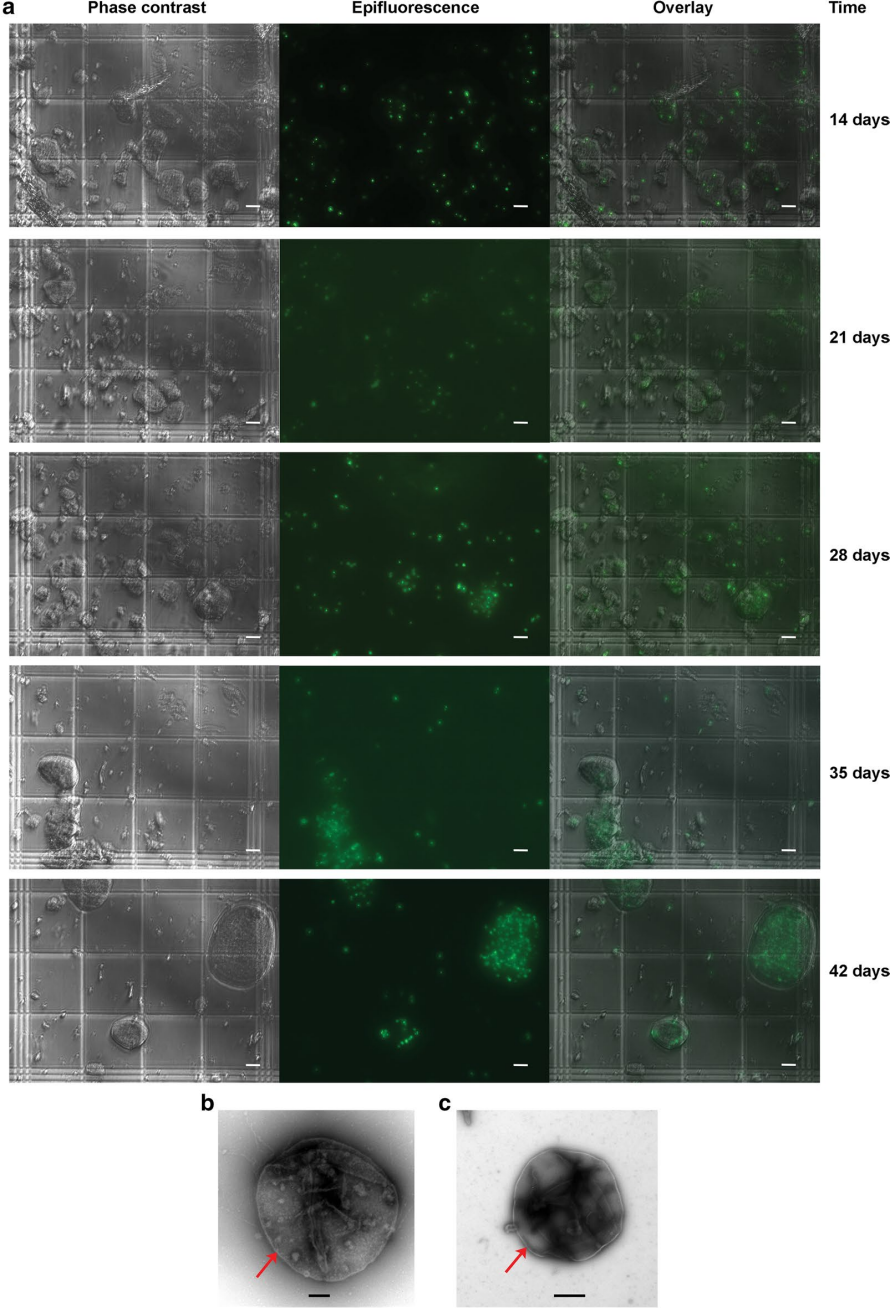
Biofilm growth of ANME-1 Mcr-producing *M. acetivorans* on methane. **a** Representative hemocytometer views (×400 magnification) of *M. acetivorans*/pES1-MAT*mcr*3 cells growing as biofilms on the precipitates after incubation on methane and 10 mM FeCl_3_. All cultures were stained with SYTO9 dye for microscopic examination. Under epifluorescence settings, cells appear green but abiotic precipitates do not fluoresce. *Scale bars* 20 µm. **b** Electron micrograph of irregular cocci *M. acetivorans*/pES1-MAT*mcr*1 growing on methane and 0.1 mM FeCl_3_. *Scale bar* 0.2 µm. **c** Electron micrograph of irregular-cocci *M. acetivorans* grown on methanol. *Scale bar* 0.5 µm. *Red arrows* show the mono-layered cell wall

Of the tested electron acceptors, the best growth on methane was seen with 10 mM Fe^3+^. After 6 weeks of incubation using low-density inocula, growth of *M. acetivorans*/pES1-MAT*mcr*3 was observed in conjunction with 9 ± 1 % of methane consumption (corresponding to 109 ± 12 µmol of methane). Critically, cell growth was evident from steadily increasing total protein levels (Fig. 2a) and from the increase in the number of cells associated with the iron precipitates (Figs. 2b, 3a). Note that the cells were differentiated from solid precipitates via SYTO9 staining, and the morphology of cells grown on methane and 0.1 mM Fe^3+^ (Fig. 3b) is comparable with the unengineered strain *M. acetivorans* grown on methanol (Fig. 3c). Up to 97 % of cells were closely associated with salt precipitates (instead of being in suspension), which suggests that these cells are growing as a biofilm on the precipitates in order to transfer electrons to the oxidized form of iron (Fig. 3a). We observed ~fivefold increase in *M. acetivorans*/pES1-MAT*mcr*3 cells on precipitates over 6 weeks of incubation (Figs. 2b, 3a); however, there was only a small two-fold increase in planktonic cell numbers. No significant changes were observed for total protein concentrations and cell counts for either cells harboring an empty plasmid or cells with pES1-MAT*mcr*3 incubated with nitrogen (changes for cell numbers on precipitates when under a nitrogen headspace were negligible after 70 days of incubation).

The identity of *M. acetivorans* during growth on methane was confirmed by 16S ribosomal DNA (rDNA) sequencing (via PCR amplification using archaeal 16S gene-specific primers). No PCR product was obtained when bacterial 16S gene-specific primers were used, providing further evidence for homogeneity of the culture. Furthermore, genome sequencing of the engineered strain that grew on methane confirmed the strain. Also, the plasmid pES1-MAT*mcr*1 was not lost from the population after 30 days of growth on methane, as we could PCR-amplify ANME-1 *mcrA* from the plasmid of the methane-grown culture (Additional file 1: Figure S3).

These results indicate that we have metabolically engineered *M. acetivorans* to grow in the presence of methane and an electron acceptor, and that the ANME-1 Mcr is necessary for this growth. Growth on methane with ferric iron is reasonable, with one example reaction for a natural consortia from marine methane-seep sediment in the Eel River Basin in California [19] presented as CH_4_ + 8 Fe(OH)_3_ + 15 H^+^ → HCO_3_^−^ + 8 Fe^2+^ + 21 H_2_O, which is thermodynamically favorable ($\Delta {\text{G}}_{\text{rxn}}^{ \circ }$ = −270 kJ/mol).

### Confirmation of growth on methane using ^13^CH_4_

Using a high inoculum size, acetate was detected extracellularly in methane-grown cultures expressing ANME-1 *mcr* under P_mcr_ANME-1_ after 5 days. Acetate concentrations were the highest for cultures producing ANME-1 Mcr from P_mcr_ANME-1_ [10.3 ± 0.8 mM corresponding to 52 ± 4 µmol, compared to 6.1 ± 0.8 mM produced by cultures with empty vector pES1(Pmat)] among all tested strains (Fig. 1c). The use of higher inoculum size also revealed low amounts of extracellular formate and pyruvate in cells with P_mcr_ANME-1_; however, these amounts were not significantly different from the other tested strains. Therefore, we concluded that acetate was probably the main product of anaerobic activation of methane in our engineered strain.

**Fig. 4:**
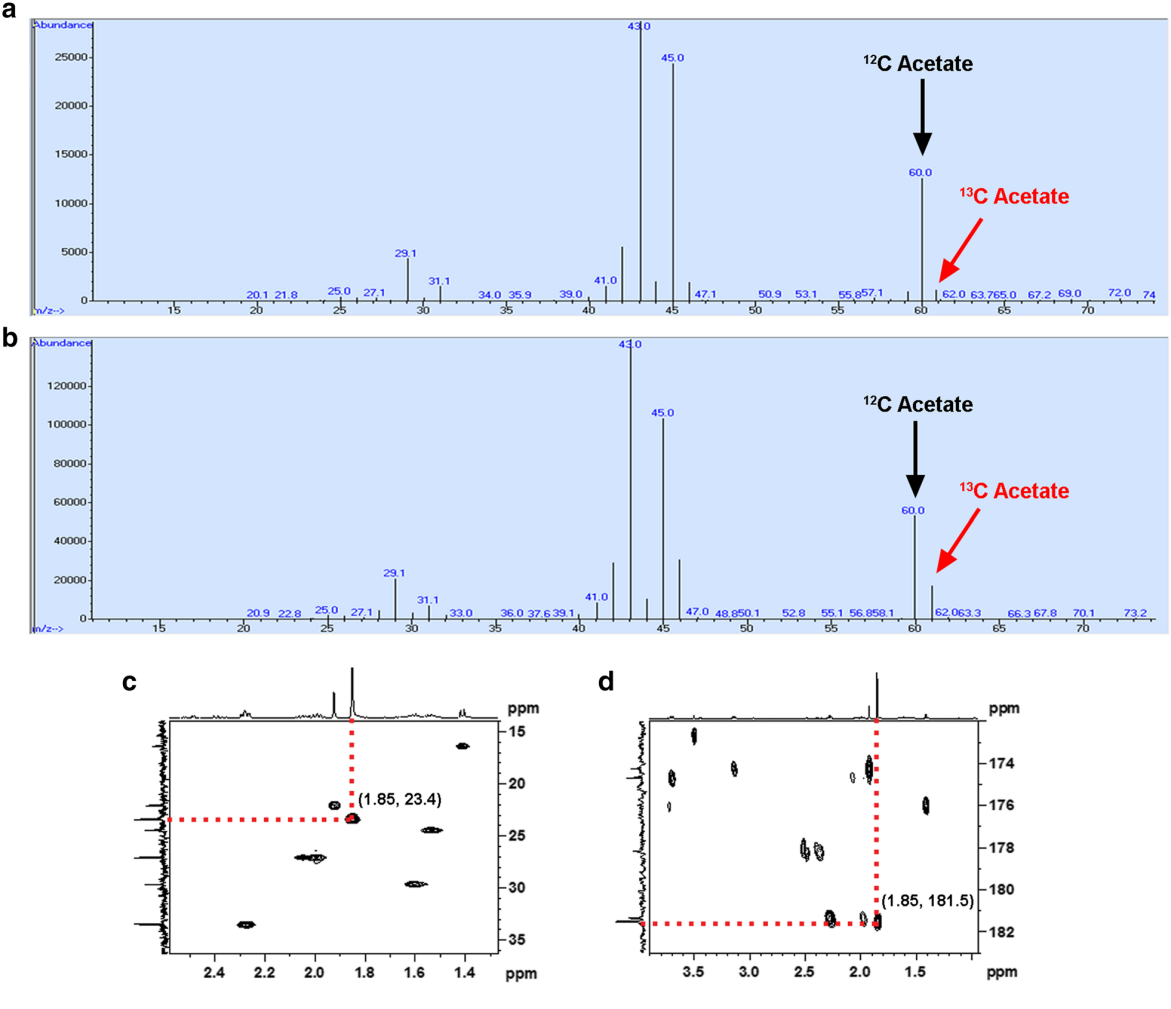
Extracellular production of acetate from ^13^CH_4_ in ANME-1 Mcr-producing *M. acetivorans* via GC/MS and NMR. **a** and **b** show GC/MS spectra of culture supernatants used to identify acetate from ^13^CH_4_. Unlabeled acetate (^12^C Acetate, *black arrows*) has a molecular mass of 60, whereas ^13^C-labeled acetate (^13^C Acetate, *red arrows*) has a molecular mass of 61. **a** *M. acetivorans* harboring empty plasmid pES1(Pmat) grown on ^13^C-labeled methane (^13^CH_4_) for 10 days. **b** *M. acetivorans* harboring pES1-MAT*mcr*3 grown on ^13^CH_4_ for 10 days. Note that the *y-axes* differ for **a** and **b**. **c** NMR HSQC spectrum identifying acetate by showing the one bond correlation between the methyl protons and the methyl carbon of acetate (^13^ CH_3_-C). **d** NMR HMBC spectrum identifying acetate in an independent manner by showing the two bonds correlation between the methyl protons and the carboxylic carbon of acetate (^13^CH_3_-COO). The *x-axes* denote ^1^H NMR plots, whereas the y-axes denote ^13^C NMR plots

To demonstrate absolutely that the methane was utilized for growth and acetate formation, ^13^CH_4_ was used as the main carbon source and the acetate product was identified three ways, by one-dimensional and multi-dimensional nuclear magnetic resonance (NMR) and by gas chromatography coupled with mass spectroscopy (GC/MS). ^13^CH_4_ incorporation was confirmed by a threefold higher ^13^C/^12^C ratio of acetate in ANME-1 Mcr-producing cells (a ^13^C/^12^C ratio of 0.33) in comparison to cells without ANME-1 Mcr (a ^13^C/^12^C ratio of 0.11) (Fig. 4a, b), and the structure of acetate confirmed by NMR (Fig. 4c, d). Our results are consistent with previous results based on trace utilization of methane [13], in which ^13^C-labeled methane is converted to acetate in *M. acetivorans*.

### Confirmation of growth on methane using ^13^C-labeled bicarbonate

We found that 19 ± 9 µmol of bicarbonate from the growth medium (Additional file 1: Table S1, 5 mL) was consumed in high-density cultures with ANME-1 Mcr produced from P_mcr_ANME-1_ showing a net methane consumption of 81 ± 16 µmol (Fig. 1a) after normalization with cells harboring an empty plasmid. Therefore, we hypothesized that bicarbonate is utilized along with methane to form acetate as has been seen for trace utilization of methane [13]. Corroborating this hypothesis, when high-density cultures of *M. acetivorans*/pES1-MAT*mcr*3 were incubated with ^13^C-labeled bicarbonate and 10 mM FeCl_3_ (with methane in the headspace), we found ^13^C incorporation into acetate using NMR and GC/MS (Additional file 1: Figure S4), which was consistent with our expectation.

Metabolic flux analysis (MFA) using ^13^C-labeled bicarbonate as a tracer was performed to quantify metabolite flows through the methanogenesis pathways resulting in the observed labeling distribution of acetate (Additional file 1: Figure S5). MFA revealed that the higher abundance of the m/z 60 ion of acetate was due to the presence of a largely unlabeled CO_2_ pool arising from the complete oxidation of unlabeled methane to CO_2_ via the methylotrophic pathway. The reduction of CO_2_ by Cdh incorporates the labeled bicarbonate into the carbonyl carbon of acetate, which is reflected in the m/z 61 ion. The abundance of m/z 62 ion of acetate can only arise from the incorporation of labeled bicarbonate into the methyl group of acetate (the 62 ion peak is small but present at higher levels than the background noise). MFA revealed a non-zero reverse flux through the methylotrophic pathway, thereby allowing the reduction of ^13^C-bicarbonate to the methyl group of acetate. In addition to this, MFA estimated the in vivo co-utilization ratio of bicarbonate/methane to be 0.06 mol/mol based on the extent of labeling of acetate; hence, complete oxidation of methane to CO_2_ via the methylotrophic pathway (v3, Additional file 1: Figure S5) produces an unlabeled CO_2_ pool resulting in a large unlabeled fraction of acetate, as seen in the ^13^C-labeled bicarbonate experiment for growth on methane (Additional file 1: Figure S4).

Although cysteine could be another carbon source (initial concentration of 3.2 mM or an amount of 16 µmol, Additional file 1: Table S1), cysteine consumption in all tested strains ranged from 32 to 49 %, with no significant differences between ANME-1 Mcr-producing strains and the strain harboring an empty plasmid. Methyl sulfides were proposed as products of AOM [20], but there is not enough sulfur in the system for methyl sulfides to be generated and thermodynamically account for significant amounts of methane consumption. We do not consider trace vitamins in the growth medium as major carbon sources, as their concentration is insignificant (less than 0.005 mM, Additional file 1: Table S1).

### Confirmation of growth on methane via iron reduction

Growth seen at 10 mM Fe^3+^ (Figs. 2, 3a) suggests the following thermodynamically possible ($\Delta {\text{G}}_{\text{rxn}}^{ \circ }$ = −325 kJ/mol) stoichiometry with Fe^3+^ as the terminal electron acceptor shown in Eq. (1).

1 $$  4\;{\text{CH}}_{4} \; + \;2\;{\text{HCO}}_{3}^{ - } \; + \;8\;{\text{Fe}}^{{3 + }} \; \to \;3\;{\text{CH}}_{3} {\text{COO}}_{{}}^{ - } \; + \;8\;{\text{Fe}}^{{2 + }} \; + \;9\;{\text{H}}^{ + }  $$

Our results using ^13^C-labeled methane and bicarbonate (Fig. 4; Additional file 1: Figure S4) demonstrate that methane and bicarbonate are converted to acetate as indicated in equation [1]; therefore, we investigated whether iron is reduced as well in long-term growth experiments. In *M. acetivorans*/pES1-MAT*mcr*3 cultures grown on methane and 10 mM FeCl_3_, 19 ± 1 µmol of Fe^3+^ were reduced into Fe^2+^ after 35 days of incubation. No other terminal electron acceptors exist in the liquid phase in significant quantities for the observed methane consumption. Hence, growth on methane and bicarbonate proceeds via iron reduction, which is consistent with cells growing as a biofilm on the FeCl_3_ precipitates (Figs. 2b, 3a).

### Genome-scale metabolic modeling

**Fig. 5:**
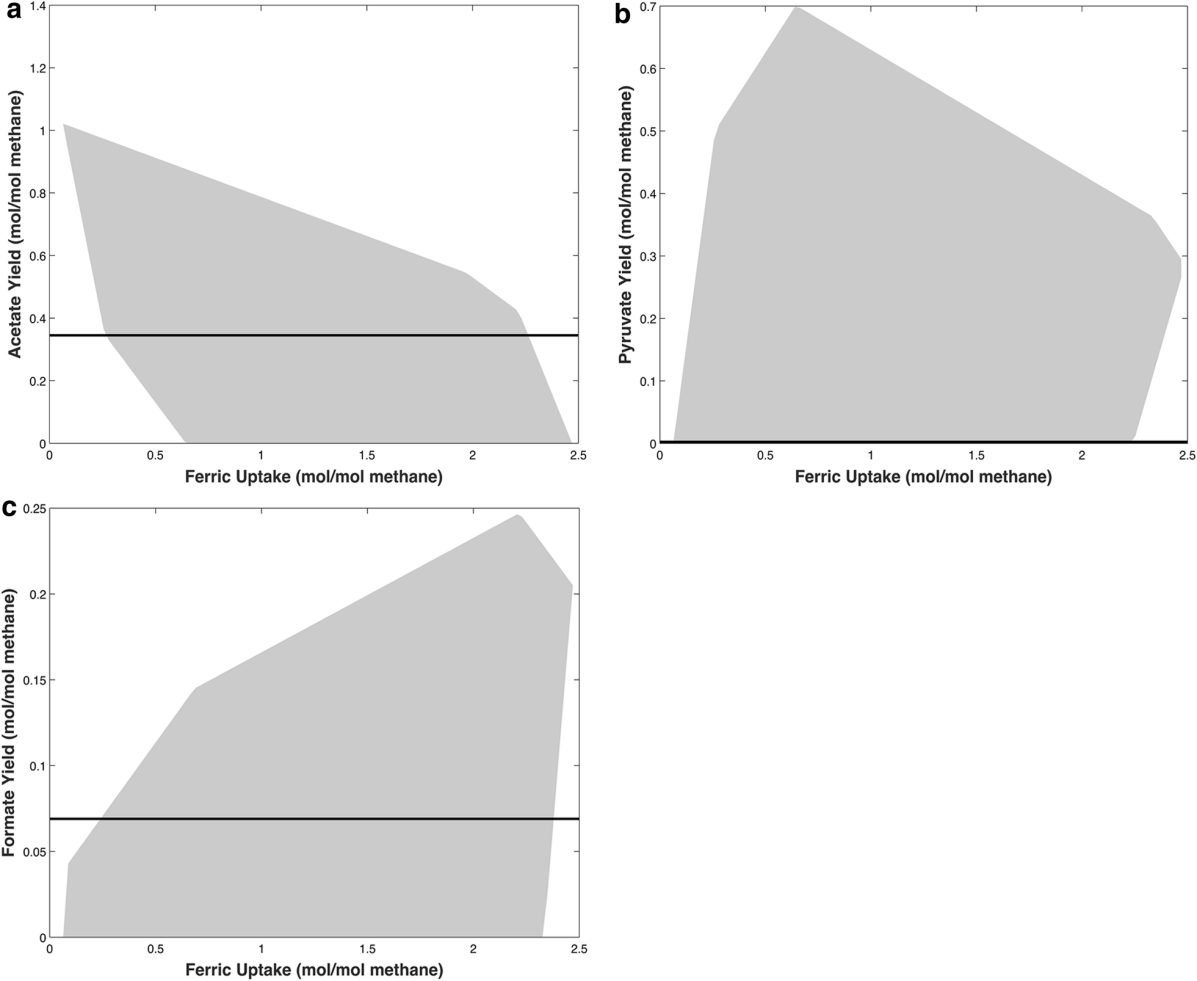
Thermodynamically feasible envelopes for the production of **a** acetate, **b** pyruvate, and **c** formate as a function of consumed Fe^3+^ as the external electron acceptor. *Horizontal lines* denote the measured amount of acetate and formate, respectively. *Both lines* intersect with the thermodynamically feasible envelope suggested by the model. Acetyl-CoA competes with iron for electrons, thus resulting in a reduction of maximum acetate production with increasing Fe^3+^ consumption. At lower Fe^3+^ consumption, the electron transport chain is not sufficient to meet the required ATP demands, thus making acetate production essential. Increasing Fe^3+^ uptake increases the flux through the methylotrophic, and consequently, the electron transport chain. At a Fe^3+^ uptake of 0.28 mol/mol methane, thermodynamic limitations restrict pyruvate production, thereby resulting in a reduced sensitivity of minimum acetate to Fe^3+^ uptake. A Fe^3+^ uptake of 0.6 mol/mol methane is sufficient to meet the ATP demands using the electron transport chain alone, thus decreasing the minimum required acetate production to zero. Since CO_2_ was not observed as a secreted product, the end product of the methylotrophic pathway was hypothesized to be formate produced by abiotic deformylation of formylmethanofuran, which competes with acetate and pyruvate for carbons causing the maximum pyruvate production to decline when Fe^3+^ uptake surpasses 0.6 mol/mol methane. However, the minimum formate production still remains zero due to the fact that formate can be oxidized to produce CO_2_ for acetate and pyruvate production. Beyond a Fe^3+^ uptake of 2.3 mol/mol methane, the thermodynamics of the system do not favor the production of acetate, thus making formate and pyruvate production mandatory

In further support of the overall reaction shown in equation [1], a recently updated genome-scale reconstruction of *M. acetivorans* (manuscript in preparation) indicates a thermodynamically feasible phenotype of co-metabolizing methane and bicarbonate using Fe^3+^ as the terminal electron acceptor. One of the possible metabolic outcomes describing the production of only acetate follows the overall stoichiometry shown in equation [1]. Alternative products supported by the metabolic model include pyruvate and formate. The thermodynamic feasibility constraints implied by the rate of production of products (acetate, pyruvate, and formate) and the rate of Fe^3+^ oxidation is described in Fig. 5. For the genome-scale reconstruction analysis, we assumed that there is no cellular growth and ATP production is only used for maintenance requirements. We found that acetate and pyruvate compete with Fe^3+^ for electrons by reductive carboxylation of methanogenesis intermediates. Pyruvate also competes with acetate production, by lowering ATP production through substrate-level phosphorylation. Therefore, the engineered cells grow on methane and bicarbonate as indicated in equation [1]. It is interesting to note that the thermodynamic infeasibility of direct reversal of the aceticlastic pathway is overcome by coupling acetate production with oxidation of methane to CO_2_ via the methylotrophic pathway.

**Fig. 6:**
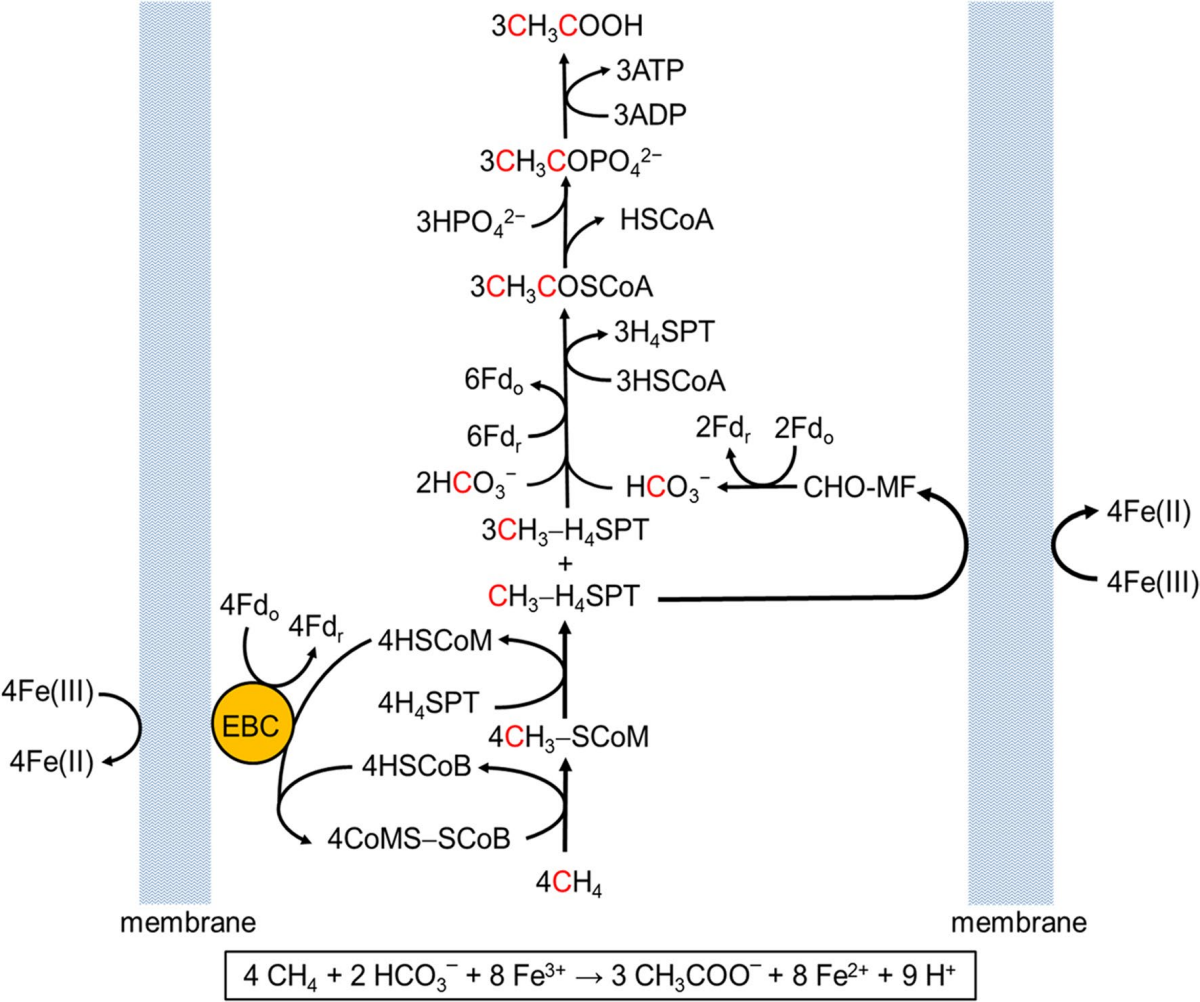
Proposed reversal of the aceticlastic pathway. The pathway shown imposes Fe^3+^ at a concentration producing the maximum amount of acetate (see Fig. 5). *Red coded atoms* indicate ^13^C-labeling. *EBC* electron bifurcating complex, *Fd*_*r*_ reduced ferredoxin, *Fd*_*o*_ oxidized ferredoxin, *CH*_*3*_ *-SCoM* methyl-coenzyme M, *HSCoM* coenzyme M, *HSCoB* coenzyme B, *CoMS-SCoB* heterodisulfide of coenzyme M and coenzyme B, *H*_*4*_ *SPT* 5,6,7,8-tetrahydrosarcinapterin, *CH*_*3*_ *-H*_*4*_ *SPT* methyl-H_4_SPT, *HSCoA* coenzyme A, *CHO-MF* formyl methanofuran, *CH*_*3*_ *COSCoA* acetyl-CoA, *HCO*_*3*_^*−*^ bicarbonate ion, *HPO*_*4*_^*2−*^ hydrogen phosphate ion, *CH*_*3*_ *COPO*_*4*_^*2−*^ acetyl phosphate, *ATP* adenosine triphosphate, *ADP* adenosine diphosphate, and *CH*_*3*_ *COOH* acetic acid

The likely biochemical pathway for the overall reaction shown in equation [1] and the basis for the genome-scale model are shown in Fig. 6. The oxidation of CH_4_ yielding CH_3_-SCoM is catalyzed by ANME-1 Mcr. The methyl group is transferred to H_4_SPT (tetrahydrosarcinapterin) by reversal of the reaction catalyzed by cytoplasmic methyltransferase (Cmt) [21] or membrane-bound methyltransferase (Mtr) [22] of methane-producing pathways. In the proposed pathway, Mtr is driven by a sodium gradient supported by oxidation of CH_3_-H_4_SPT (methyl-H_4_SPT) to CO_2_ and reduction of Fe^3+^ that generates a proton gradient exchanged for sodium (not shown). The CH_3_COSCoA (acetyl-CoA) is a product of the reversible Cdh complex [23, 24]. The carbonyl group of CH_3_COSCoA is derived from the ferredoxin-dependent [25] reduction of exogenous and endogenous bicarbonate derived from oxidation of the methyl of CH_3_-H_4_SPT consistent with the ^13^C labeling pattern (red colored carbon atoms). Two molecules of reduced ferredoxin derive from oxidation of CHO-MF (formyl methanofuran) by formyl methanofuran dehydrogenase [26]. The remaining reduced ferredoxins derive from oxidation of HSCoB and HSCoM (coenzyme M) catalyzed by a hypothesized electron transport system bypassing reversal of the membrane-bound system in the forward pathway dependent on a sodium gradient [27]. This system is hypothesized to involve an electron bifurcating complex (EBC), which transfers electrons from the oxidation of HSCoM and HSCoB to a membrane-bound electron carrier reducing Fe^3+^. The EBC is postulated to couple the thermodynamically unfavorable reduction of ferredoxin (*E*_m_ = −420 mV) to the favorable reduction of Fe^3+^ (*E*_m_ = +770 mV). Importantly, the system is postulated to drive the unfavorable oxidation of CH_4_ and transfer of the methyl group to H_4_SPT. The CH_3_COSCoA is converted to acetate by the reversible phosphotransacetylase and acetate kinase that produces ATP, supporting growth with CH_4_ as the energy source.

We also used RNA sequencing to determine differential gene expression in methane-grown cells producing ANME-1 Mcr by P_mcr_ANME-1_ in comparison to the same cells grown on methanol. Two genes were induced by growth on methane (Additional file 1: Table S2): MA1997 (hypothetical protein; absent in methanol-grown cells) and MA0463 (ferredoxin; tenfold induction via methane). Critically, 27 genes were repressed by growth on methane, including genes specific for methanogenesis (6- to 19-fold repression) such as *mcrA*, *mcrB*, *mtaB 1*, and *mtaB 2* (Additional file 1: Figure S6). Altogether, since the methane-generating pathway from carbon dioxide was repressed (e.g., host *mcrAB, mtaB 1B2, cdhB, fmdD*) (Additional file 1: Figure S6), growth on methane appears to proceed through reverse methanogenesis.

## Conclusions

By producing Mcr from ANME-1 in a methanogenic host, we are able to reverse the rest of the methanogenic pathway. This work thus provides evidence indicating the importance of AOM in the methane cycle as AOM converts methane into acetate as one byproduct. Results from these laboratory-scale experiments show that reversing methanogenesis via ANME-1 Mcr production is a promising anaerobic technology to oxidize methane for biofuel production as well. Depending on the type of ANME populations and their syntrophic partners, natural AOM can proceed via reduction of iron, manganese, or sulfate [7, 19]. However, with our engineered *M. acetivorans*/pES1-MAT*mcr*3 cells, growth on methane was possible only via iron reduction, which highlights the distinctiveness of our engineered population of *M. acetivorans*/pES1-MAT*mcr*3 cells.

*M. acetivorans* is an excellent host for biofuel production due to its inherent tolerance to alcohol (i.e., growth on 150 mM methanol). Furthermore, acetate from our engineered strain can be used to produce ethanol and other types of biofuels; for example, fatty alcohols, alkanes, and acyl esters through acetyl-CoA and farnesane, farnesanol, and isopentanol through the isoprenoid pathway. The culturability of our engineered strain also renders it amenable to further engineering efforts to biologically capture methane and potentially convert it to usable resources. Overall, our engineered strain opens up possibilities for efficient production of acetate that can be scaled up for industrial uses and provides a host that allows active Mcr from ANME-1 to be studied for the first time.

## Methods

### Growth conditions of *M. acetivorans*

All *M. acetivorans* strains (Additional file 1: Table S3) were routinely grown anaerobically as pre-cultures at 37 °C in an 80 % N_2_/19 % CO_2_/1 % H_2_ atmosphere with mild shaking in 10 mL HS medium [28] with 150 mM methanol as the carbon source. Cell growth was measured spectrophotometrically and direct cell counts were confirmed by staining cell cultures (often containing precipitates) with SYTO9 dye (Life Technologies, Carlsbad, CA, USA) and viewed microscopically using a bright-line hemocytometer (Hausser Scientific, Horsham, PA, USA) under phase-contrast and epifluorescence settings (Zeiss Axio Scope.A1, Germany). All 28-mL culture tubes (18 × 150 mm, Bellco Glass, Vinelanad, NJ, USA) were sealed by aluminum crimp seals. Plasmids were maintained with 2 µg/mL puromycin.

For long-term (ca., 40 days) growth on methane with low-density inocula (1 %), the *M. acetivorans* strains were grown in 5 mL HS medium in 28-mL culture tubes with additional electron acceptors at 37 °C with mild shaking; the electron acceptors tested were FeCl_3_, FeSO_4_, KNO_3_, NaNO_3_, NaNO_2_, and ZnSO_4_ (0.05–100 mM) as well as Fe_2_O_3_ (1 mM) and MnO_2_ (2 mM). As a frame of reference, a concentration of 0.1 mM would be 0.5 µmol in a 5-mL culture. The preferred carbon sources of *M. acetivorans*, methanol, trimethylamine, and acetate, were not present in the medium. The headspace of the tube was filled with methane (99.999 % purity, catalog no. ME5.0RS, Praxair, Danbury, CT), and crimped with aluminum seals. PCR was used to verify the presence of pES1-MAT*mcr*1 in methane-grown cells after 30 days of incubation. Cells were used as genomic templates for PCR amplification of ANME-1 *mcrA* using primers B4-f and pES1-r (Additional file 1: Table S4). Cell morphology was examined on a transmission electron microscope (FEI Tecnai G2 Spirit BioTwin, Hillsboro, OR, USA) using uranyl acetate-stained cells.

To document cell growth and methane consumption as a function of time, 10^7^ CFU/mL of *M. acetivorans*/pES1-MAT*mcr*3 and *M. acetivorans*/pES1(Pmat) cells were incubated at 37 °C for 6 weeks in 8 mL HS medium and 10 mM FeCl_3_ in 40-mL bottles stoppered with butyl rubber stoppers and crimped with aluminum seals. Each culture and its methane in the headspace were sampled every 1–2 weeks. After each sampling, petroleum jelly was applied to the surface of the stoppers to prevent leaking from needle punctures. All culture bottles were inverted during incubation to prevent methane from escaping from the vessels, and were shaken to ensure homogenous mixing of liquid, precipitates, and cells. As a control, cultures grown on methanol in parallel reached a saturated density of (4 ± 0.3) × 10^8^ CFU/mL.

For short-duration (ca., 5 days) growth on methane in which high cell-density inocula were used, 2 mL of each strain was pre-grown in 200 mL of HS medium with 150 mM methanol (and 2 µg/mL puromycin when plasmids were present) at 37 °C for 5 days (OD_600_ ~ 1.0). Cells were collected by centrifugation (5000 rpm for 20 min), and were washed three times with HS medium and puromycin alone to remove residual methanol. The final cell pellet was resuspended using 5 mL of HS medium supplemented with 0.1 or 10 mM FeCl_3_ and 2 µg/mL puromycin when appropriate, to yield a density of 4 × 10^10^ CFU/mL. After filling the headspace of each tube with methane, the tubes were incubated at 37 °C with mild shaking for 5–10 days.

### Cloning ANME-1 *mcrBGA*

All oligonucleotides are listed in Additional file 1: Table S4. The ANME-1 *mcrBGA* genes (3.9 kb, locus tag fos0113c9_0022-0024, Genbank accession FP565147.1) encoding the ANME-1 Mcr whose 3D structure has been determined [15], was assembled from six DNA fragments of 600–700 bp (Integrated DNA Technologies) using the Gibson assembly method [29]. Unlike the *mcr* locus from *M. acetivorans* [16] and other methanogenic archaea [30], *mcrC* and *mcrD* are not present. ANME-1 *mcrBGA* were cloned downstream of promoter P_cdh_ using the XbaI and BmtI sites of pES1 to form pES1-MAT*mcr*1. After electroporating the plasmid into *E. coli* DH5α-λ*pir,* the complete *mcrBGA* locus and promoter region were sequenced (via primers veri-p-f, pES1-f, MATmcrB2-f, MATmcrB3-f, MATmcrB4-r, and pES1-r) to confirm no errors were introduced during cloning. For pES1-MAT*mcr*2, in which ANME-1 *mcrBGA* genes are under the transcription of *mcr* promoter from *M. acetivorans* (P_mcr_*M. acetivorans*_), a 419-bp DNA fragment that corresponds to P_mcr_*M. acetivorans*_ was amplified from the genomic DNA (catalog #35395D-5, American Type Culture Collection, Manassas, VA, USA) using primers Pmcr-f2 and Pmcr-r2. P_mcr_*M. acetivorans*_ is further fused to ANME-1 *mcrBGA* using overlap PCR via primers Pmcr-f2 and B6-r1. To place ANME-1 *mcrBGA* under control its native promoter (which we named P_mcr_ANME-1_), a 237-bp DNA fragment upstream of the ANME-1 *mcrBGA* genes was synthesized and assembled with *mcrBGA* to create P_mcr_ANME-1_::*mcr*_ANME-1_ using overlap PCR (via primers Pmat-f, Pmat-r, and B6-r1). The resulting plasmid is pES1-MAT*mcr*3. The empty plasmid harboring P_mcr_ANME-1_ was created by linearizing the vector backbone of pES1-MAT*mcr*3 using primers pES1(Matprom)-f and pES1(Matprom)-r, followed by self-ligation of the backbone to form pES1(Pmat). All plasmids were transformed into *M. acetivorans* using liposome-mediated transformation [31].

### Genome sequencing

Genomic DNA was isolated using the Ultraclean^®^ Microbial DNA Isolation Kit (MO BIO Laboratories, Carlsbad, CA, USA). After shearing the genomic DNA using a Covaris ultrasonicator, DNA fragments were barcoded using TruSeq DNA Nano (Illumina, San Diego, CA, USA). The pooled, barcoded DNA library was sequenced on a MiSeq sequencing platform (Illumina) to generate 2,597,170 paired-end reads of 150 bp for *M. acetivorans* (ancestral strain) grown on methanol, and 2,699,253 paired-end reads of 150 bp for *M. acetivorans*/pES1-MAT*mcr*1 grown on methane and 0.1 mM FeCl_3_.

To identify single-nucleotide polymorphisms, insertions, and deletions between the ancestral strain and the methane-grown strain, sequencing reads of each strain were mapped to the reference genome of *M. acetivorans* (Genbank accession NC_003552.1) using the Burrows-Wheeler Alignment tool [32]. Aligned reads were sorted based on mapped position in the reference genome using SortSam from the Picard tools (http://broadinstitute.github.io/picard). Misalignments caused by insertions and deletions were corrected locally using IndelRealigner from the Genome Analysis ToolKit package [33]. Duplicated reads were marked using MarkDuplicates.jar from the Picard tools to remove sequencing bias. Unified Genotyper [33] was then used to call the variants of each strain after removing variants that are present in less than 40 % of the population. The differences between the ancestral strain and the methane-grown strain were identified using vcftools [34].

### 16S rDNA amplification and sequencing

To verify archaeal strains, primers ARCH109-F and ARCH934-R [35] were used to amplify an 0.8-kb PCR product of 16S rDNA genes. To verify the absence of bacteria, primers 27F and 1492-R [36] were used to amplify an 1.5-kb PCR product of 16S rDNA genes.

### Total protein, cysteine, bicarbonate, and iron reduction assays

Cultures (120 µL) were centrifuged briefly to remove the supernatant, and the cell pellets were resuspended with 10–24 µL of sterile water to lyse the cells. The total protein concentration of these cell suspensions was determined using the Bradford assay (Bio-Rad Laboratories, Hercules, CA, USA).

The concentration of total cysteine (reduced and oxidized forms) in HS media (in which cells showed methane consumption) was determined spectrophotometrically using ninhydrin which reacts specifically with l-cysteine, even in the presence of other thiols, to form a pink-colored product (ε_max_ 560 nm) under acidic conditions [37]. The concentration of bicarbonate of HS media (in which cells showed methane consumption) was measured spectrophotometrically using the MaxDiscovery™ Carbon Dioxide Enzymatic Assay Kit (Bioo Scientific, Austin, TX, USA). Filtered supernatant of each culture was used as a sample solution.

The reduction of iron was measured by employing the ferrozine method for quantification of only reduced iron in the form of Fe^2+^, as described previously [38]. A 100-µL of culture was immediately mixed with 33 µL of 2 N HCl. Then, 10 µL of the acidified mixture was mixed with 190 µL of 1 mg/mL ferrozine in 100 mM HEPES (pH 7.0), and absorbance at 562 nm was measured. Actual Fe^2+^ concentrations were calculated by comparing results with those from standard solutions of Fe^2+^.

### Gas chromatography (GC) and high-performance liquid chromatography (HPLC)

GC analyses were conducted for quantifying methane in the culture headspace. Aliquots of 50 or 100 µL volumes were passed through a 6890 N Agilent gas chromatograph equipped with a 60/80 Carboxen-1000 column (4600 × 2.1 mm, Supelco catalog no. 12390-U) and a thermal conductivity detector. The injector, column, and detector were maintained at 150, 180, and 240 °C, respectively. Carrier gas flow (nitrogen) was kept at 20 mL/min, and reference gas flow (also nitrogen) for the detector at 20 mL/min as well. Gases were identified according to their retention times and their concentrations were determined according to comparisons with standards.

HPLC analyses were conducted for the detection and quantification of three organic acids under investigation (acetic acid, formic acid, and pyruvic acid). All samples were filtered through a 0.22 µm polyvinylidene fluoride membrane before diluting 1:6 in running buffer (0.0025 M sulfuric acid in water), then 60 µL of the 1:6 dilution was fractionated by HPLC (Waters 717 autosampler with a model 515 pump, and a 2996 photodiode array detector) with a reversed-phase column [Phenomenex Rezex ROA-Organic Acid H + (8 %) (300 × 7.8 mm)]. Separations were conducted using an isocratic flow rate of 0.4 mL/min 0.0025 M sulfuric acid in water. Absorbance at 210 nm was used to detect all compounds. Chemicals used as standards for comparisons are glacial acetic acid (EMD Millipore, catalog no. AX0073-6), sodium formate (catalog no. BP356-100, Fisher Scientific, Hampton, NH, USA), and sodium pyruvate (catalog no. S648-500, Fisher Scientific). Peaks corresponding to those of pyruvic acid, formic acid, and acetic acid were confirmed by retention time, co-elution with standards, and by comparing absorbance spectra with those from the standards. Total quantities of the compounds were calculated by comparing peak areas with standard curves made by running chemical standards.

### Western blot

To demonstrate production of ANME-1 Mcr, a FLAG epitope tag was introduced into the carboxy terminus of ANME-1 McrA encoded by *mcrA* in pES1-MAT*mcr*1, pES1-MAT*mcr*2, and pES1-MAT*mcr*3. The DNA encoding the FLAG tag was incorporated into primer B6-r-flag, which was used along with the respective forward primers to create ANME-1 *mcrA*-*flag*. After transformation, *M. acetivorans* harboring pES1-MAT*mcr*1-*flag*, pES1-MAT*mcr*2-*flag*, and pES1-MAT*mcr*3-*flag* were grown on 200 mL HS-methanol for 5 days, and used for short-duration growth experiments. Methane consumption was measured after 5 days, and cells were harvested by centrifugation. Each cell pellet was resuspended in 2 mL Lysis Buffer [20 mM Tris–HCl, 0.1 mM EDTA, 500 mM ε-aminocaproic acid, 10 % glycerol, 1 µL protease inhibitor cocktail (Sigma)]. Cells were sonicated on ice at a power level of 10 for 150 s (30 cycles of 5 s each, 60 Sonic Dismembrator, Fisher Scientific). Total proteins were resolved via 12 % Tris–glycine-SDS gels. Western blots were performed with monoclonal horseradish peroxidase-conjugated antibodies raised against a FLAG epitope tag (Thermo Scientific, Waltham, MA, USA). Blotted proteins were detected using the chemiluminescence reagents from the SuperSignal West-Pico Chemiluminescence kit (Thermo Scientific).

### ^13^C-labeled methane-grown cultures, ^13^C-labeled bicarbonate-grown cultures, ^13^C NMR, and GC/MS

Starter cultures (200 mL) of *M. acetivorans*/pES1(Pmat) and *M. acetivorans*/pES1-MAT*mcr*3 were used for short-duration growth experiments. For cultures incubated with ^13^C-labeled methane, the headspace was filled with ^13^C-labeled methane (99 % ^13^C atom, Sigma). HS medium for cultures incubated with ^13^C-labeled bicarbonate was prepared using ^13^C-labeled sodium bicarbonate (99 % ^13^C atom, Cambridge Isotope Laboratories, Tewksbury, MA, USA). All cultures were incubated at 37 °C for 10 days. Acetate was measured using an Agilent 7890A/5975C GC/MSD using a Nukol (Supelco) capillary column (30 × 0.32, 0.25 μm phase thickness comprised of a bonded polyethylene glycol). Incorporation was determined by integrating the peaks areas corresponding to ^12^C and ^13^C acetate. NMR experiments were conducted on a Brüker Avance III HD spectrometer operating at 500.20 MHz and 125.78 MHz for ^1^H and ^13^C nuclei, respectively, using H_2_O:D_2_O as a solvent. ^13^C and DEPT-135 spectra were recorded with a spectral width of 220 ppm, using 64 K data points, a 90° excitation pulse (11 µs) and relaxation delay of 5 s. 1 k scans were collected and spectra zero-filled to 128 K. For all FIDs, line broadening of 1 Hz was applied prior to Fourier transform. Chemical shifts are reported in ppm from DSS (*δ* = 0). The gradient-selected ^1^H-^13^C heteronuclear multiple bond correlation (gHMBC) experiment was performed using a low-pass J-filter (3.4 ms) and delays of 65 and 36 ms to observe long-range C–H couplings with 256 increments and 64 transients of 2048 data points. The relaxation delay was 2.0 s. Zero-filling to a 2 K × 2 K matrix and π/2-shifted sine square bell multiplication was performed prior to Fourier transform. Heteronuclear Single Quantum Coherence (gHSQC) spectra were recorded with 256 increments in *F*1 and 32 scans per increment, using the standard hsqcetgpsisp.2 Brüker pulse sequence. Relaxation delay of 2 s and 2 K data points was used for spectral width of 10 ppm in the proton dimension, whereas the spectral width in the carbon dimension was 180 ppm.

### RNA sequencing

Differential gene analysis of two growth conditions (three biological replicates each) was performed: (1) *M. acetivorans*/pES1-MAT*mcr*3 contacted with methane and (2) *M. acetivorans*/pES1-MAT*mcr*3 grown on methanol. All starter cultures (200 mL) were grown on methanol for 5 days, and harvested by centrifugation. Cell pellets were washed three times with HS medium, and resuspended using 5 mL HS medium, 2 µg/mL puromycin, and 0.1 mM FeCl_3_. For condition (1), methane was filled into the headspace of the cultures. For condition (2), 150 mM methanol was added. All cultures were incubated at 37 °C for 5 days, followed by rapid centrifugation in the presence of 50 µL RNA*later* solution (Ambion, Austin, TX, USA) per mL of culture. Total RNA isolated using the RNeasy Mini kit (Qiagen, Valencia, CA, USA) was digested with terminator 5′-phosphate-dependent exonuclease (Epicentre, Madison, WI, USA) to partially remove ribosomal RNA. Digested RNA was cleaned using AgenCourt RNAClean XP beads (AgenCourt Bioscience, Beverly, MA, USA) and used for cDNA library construction using the TruSeq Stranded mRNA Library kit (Illumina). The pooled and barcoded cDNA library was sequenced on a HiSeq sequencing platform (Illumina). Obtained reads were mapped to the reference genome of *M. acetivorans* (Genbank accession NC_003552.1) and plasmid pES1-MAT*mcr*3 using STAR [39]. The mapped reads were assembled using Cufflink v2.2.1 [40] to identify potential novel transcripts. Assembled, unannotated novel transcripts for all the strains were combined with the list of known genes. Differential expression of genes and potential novel transcripts were determined using Cuffdiff [40] at a significance cutoff at *q* < 0.07 with a false discovery rate of 0.05. Expression levels of gene transcripts are expressed as fragments per kilobase of transcript per million mapped fragments (FPKM) [41], and expression changes are determined by the ratio of FPKM of culture replicates grown on methane to FPKM of culture replicates grown on methanol. Gene expression data have been deposited in the Gene Expression Omnibus under accession code GSE66445.

## Additional file

10.1186/s12934-015-0397-z This file consists of four supplemental tables and six supplemental figures. **Table S1** lists the components in the HS medium used to grow ANME-1 Mcr-producing *M. acetivorans* on methane and 0.1 mM or 10 mM FeCl_3_. **Table S2** shows the fold changes of differentially expressed genes in *M. acetivorans*/pES1-MAT*mcr*3 grown on methane and 0.1 mM FeCl_3_, in comparison to the same strain grown on methanol. **Table S3** lists the strains and plasmids, and **Table S4** lists the oligonucleotides, used in this study. **Figure S1** shows the three promoters used to express ANME-1 *mcrBGA* genes. **Figure S2** shows the detected McrA-FLAG in *M. acetivorans*/pES1-MAT*mcr*3-*flag* grown on methane after five days. **Figure S3** shows the detection of ANME-1 mcrA after 30 days of growth on methane. **Figure S4** shows the GC/MS spectra of culture supernatants used to identify acetate from H^13^CO. **Figure S5** shows the flux through the various reactions in the methanogenesis pathway of *M. acetivorans* estimated by ^13^C-metabolic flux analysis using ^13^C-labeled bicarbonate as the input tracer. **Figure S6** shows a simplified methanogenesis pathway from CO_2_ and CH_3_OH of *M. acetivorans*.

## Abbreviations

ANME-1: anaerobic methanotrophic archaeal population 1
AOM: anaerobic oxidation of methane
CH_3_-SCoM: methyl-coenzyme M (2-(methylthio)ethanesulfonate)
ATP: adenosine triphosphate
CoBS-SCoM: heterodisulfide of coenzyme B and coenzyme M
*E*_m_: resting potential
Fe^3+^: ferric ion
Fe^2+^: ferrous ion
FeCl_3_: ferric chloride
$\Delta {\text{G}}_{\text{rxn}}^{ \circ }$: Gibbs free energy change upon reaction
HS: high-salt
HSCoB: coenzyme B. CH_4_ (methane)
Mcr: methyl-coenzyme M reductase
Mn^4+^: manganese ion
NO_3_^−^: nitrate ion
NO_2_^−^: nitrite ion
P_mcr_ANME-1_: the *mcr* promoter from ANME-1
P_cdh_: the CO dehydrogenase/acetyl-CoA synthase promoter from *Methanosarcina thermophila*
P_mcr_*M. acetivorans*_: the *mcr* promoter from *M. acetivorans*
SO_4_^2−^: sulfate ion
