# Supplementary material for: Reversing methanogenesis to capture methane for liquid biofuel precursors
Source: Microb Cell Fact. 2016 Jan 14;15:11. doi: 10.1186/s12934-015-0397-z (PMC4714516; doi:10.1186/s12934-015-0397-z)
Supplement: Supplementary file 1 — 10.1186/s12934-015-0397-z This file consists of four supplemental tables and six supplemental figures. Table S1 lists the components in the HS medium used to grow ANME-1 Mcr-producing M. acetivorans on methane and 0.1 mM or 10 mM FeCl3. Table S2 shows the fold changes of differentially expressed genes in M. acetivorans/pES1-MATmcr3 grown on methane and 0.1 mM FeCl3, in comparison to the same strain grown on methanol. Table S3 lists the strains and plasmids, and Table S4 lists the oligonucleotides, used in this study. Figure S1 shows the three promoters used to express ANME-1 mcrBGA genes. Figure S2 shows the detected McrA-FLAG in M. acetivorans/pES1-MATmcr3-flag grown on methane after five days. Figure S3 shows the detection of ANME-1 mcrA after 30 days of growth on methane. Figure S4 shows the GC/MS spectra of culture supernatants used to identify acetate from H13CO. Figure S5 shows the flux through the various reactions in the methanogenesis pathway of M. acetivorans estimated by 13C-metabolic flux analysis using 13C-labeled bicarbonate as the input tracer. Figure S6 shows a simplified methanogenesis pathway from CO2 and CH3OH of M. acetivorans. [file 12934_2015_397_MOESM1_ESM.pdf]

SUPPLEMENTARY INFORMATION

Reversing Methanogenesis to Capture Methane for Liquid Biofuel Precursors

Valerie W. C. Soo[1,¶], Michael J. McAnulty[1,¶], Arti Tripathi[1], Fayin Zhu[1], Limin Zhang[2,6], Emmanuel Hatzakis[3], Philip B. Smith[5], Saumya Agrawal[7], Hadi Nazem-Bokaee[1], Saratram Gopalakrishnan[1], Howard M. Salis[1], James G. Ferry[4], Costas D. Maranas[1], Andrew D. Patterson[2], and Thomas K. Wood[1,4*]

Departments of Chemical Engineering[1], Veterinary and Biomedical Sciences[2], Chemistry[3], and Biochemistry and Molecular Biology[4], and The Huck Institutes of the Life Sciences[5], The Pennsylvania State University, University Park, Pennsylvania 16802-4400, USA. Institute of Natural and Mathematical Sciences[7], Massey University, Auckland 0632, New Zealand. Chinese Academy of Sciences Key Laboratory of Magnetic Resonance in Biological Systems[6], Wuhan Institute of Physics and Mathematics, Wuhan 430071, China.

*Corresponding author (tuw14@psu.edu)
¶These authors contributed equally.

Supplementary Table 1. Components in HS medium used to grow ANME-1 Mcr-producing *M. acetivorans* on methane and 0.1 mM or 10 mM $FeCl_3$.

Chemical	Final concentration (mM)	Amount in 5 mL (µmol)	Amount in 8 mL (µmol)
NaCl	400	2000	3200
$MgSO_4.6H_2O$	54	270	430
$NaHCO_3$	45	230	360
Methane	41	210	330
NH_4Cl	19	95	150
KCl	13	65	100
KH_2PO_4	5.0	25	40
Cysteine.HCl	3.2	16	26
$CaCl_2.2H_2O$	2.0	10	16
$Na_2S.9H_2O$	1.0	5.0	8.0
$MgSO_4.7H_2O$	0.10	0.50	0.80
$FeCl_3.6H_2O$	0.10 or 10	0.50 or 50	0.80 or 80
Nitrilotriacetic acid	0.079	0.40	0.63
$MnSO_4.H_2O$	0.030	0.15	0.24
$ZnCl_2$	0.0059	0.030	0.047
Pyridoxine hydrochloride	0.0049	0.025	0.039
Nicotinic acid	0.0041	0.021	0.033
Resazurin (sodium salt)	0.0040	0.020	0.032
p-Aminobenzoic acid	0.0037	0.019	0.030
Puromycin dihydrochloride	0.0037	0.019	0.030
$CoSO_4.7H_2O$	0.0036	0.018	0.029
$FeSO_4.7H_2O$	0.0036	0.018	0.029
Lipoic acid	0.0024	0.012	0.019
Boric acid	0.0016	0.0080	0.013
Thiamine hydrochloride	0.0015	0.0075	0.012
Riboflavin	0.0013	0.0065	0.010
Pantothenic acid (calcium salt)	0.0010	0.0050	0.0080
$Na_2MoO_4.2H_2O$	0.0010	0.0050	0.0080
$NiCl_2.6H_2O$	0.0010	0.0050	0.0080
Biotin	0.00082	0.0041	0.0066
Folic acid	0.00045	0.0023	0.0036
$CuSO_4.5H_2O$	0.00040	0.0020	0.0032
$KAl(SO_4)_2.12H_2O$	0.00021	0.0011	0.0017
Vitamin B_{12}.HCl	0.0000074	0.000037	0.000059

Supplementary Table 2. **Differentially-expressed genes in *M. acetivorans*/pES1-MAT*mcr*3 grown on methane and 0.1 mM $FeCl_3$ compared to *M. acetivorans*/pES1-MAT*mcr*3 grown on methanol, as determined by RNA sequencing.** Fold change is determined by the ratio of FPKM of three replicates grown on methane ($FPKM_{methane}$) to FPKM of three replicates grown on methanol ($FPKM_{methanol}$). A positive fold change indicates induced expression, whereas a negative fold change indicates repressed expression. Statistical significance after taking false discovery rate of 0.05 into account was determined at $q < 0.07$.

Gene	Gene product	$FPKM_{methane}$	$FPKM_{methanol}$	Fold change	Genome coordinates of mapped read
**Induced**					
MA1997	Hypothetical protein	9.2	0	∞	NC_003552.1: 2,473,596 → 2,473,934
MA0463	Ferredoxin	318.6	33.3	9.6	NC_003552.1:542,619 ← 542,799
**Repressed**					
MA3469	Hypothetical protein	16.3	3751.7	-229.1	NC_003552.1:4,280,293 ← 4,280,638
MA3300	Hypothetical protein	72.5	2104.0	-29.0	NC_003552.1: 4,075,459 → 4,075,671
novel transcript	Unannotated	15.4	433.1	-28.1	NC_003552.1:1,615,210 – 1,615,658
MA4384	Hypothetical protein	1.2	29.3	-25.1	NC_003552.1: 5,411,710 → 5,413,605
mcrA (Host)	Methyl coenzyme M reductase, subunit alpha	9.4	178.9	-18.6	NC_003552.1: 5,596,675 ← 5,598,387
mcrB (Host)	Methyl coenzyme M reductase, subunit beta	5.9	62.5	-10.7	NC_003552.1: 5,600,310 ← 5,601,614
mtaB1	Methanol-5-hydroxybenzimidazolylcobamide co-methyltransferase, isozyme 1	5.9	75.5	-12.8	NC_003552.1: 534,844 ← 536,229
mtaB2	Methanol-5-hydroxybenzimidazolylcobamide co-methyltransferase, isozyme 2	4.4	28.3	-6.4	NC_003552.1:5,420,051 → 5,421,437
novel transcript	Unannotated	0	322.5	-∞	NC_003552.1:1,440,944 – 1,441,203
novel transcript	Unannotated	0	116.7	-∞	NC_003552.1:63,866 – 64,037
atpC	V-type ATP synthase subunit C	0	24.7	-∞	NC_003552.1: 5,071,956 → 5,072,204

cdhB	Acetyl-CoA decarbonylase/synthase complex, subunit epsilon	0	13.7	-∞	NC_003552.1: 1,211,315 ← 1,211,827
fmdD	Formylmethanofuran dehydrogenase, subunit D	0	6.6	-∞	NC_003552.1: 367,095 → 367,484
groES	Co-chaperonin GroES	0	11.4	-∞	NC_003552.1: 741,925 → 742,203
MA0369	Hypothetical protein	0	6.4	-∞	NC_003552.1: 435,971 ← 436,561
MA0515	Hypothetical protein	0	19.5	-∞	NC_003552.1: 599,612 ← 600,484
MA0609	H/ACA RNA-protein complex component Gar1	0	9.5	-∞	NC_003552.1: 715,092 → 715,352
MA1027	Hypothetical protein	0	11.2	-∞	NC_003552.1: 1,228,492 → 1,228,737
MA1090	50S ribosomal protein L19	0	7.6	-∞	NC_003552.1: 1,295,017 → 1,295,472
MA1376	Phosphoribosylaminoimidazole carboxylase	0	8.6	-∞	NC_003552.1: 1,643,428 ← 1,643,823
MA1663	Hypothetical protein	0	89.3	-∞	NC_003552.1: 1,996,878 → 1,997,207
MA2558	Hypothetical protein	0	7.1	-∞	NC_003552.1: 3,183,587 ← 3,183,991
MA3479	Ferrous iron transport protein A	0	25.6	-∞	NC_003552.1: 4,305,144 ← 4,305,392
MA4194	Ferredoxin	0	92.5	-∞	NC_003552.1: 5,118,713 → 5,118,889
MA4277	50S ribosomal protein L12	0	151.1	-∞	NC_003552.1: 5,197,662 → 5,197,976
MA4508	Hypothetical protein	0	37.8	-∞	NC_003552.1: 5,555,059 → 5,555,268
MA4565	Hypothetical protein	0	690.5	-∞	NC_003552.1: 5,626,276 ← 5,626,488

Supplementary Table 3. Strains and plasmids used in this study.

Strain/Plasmid	Description	Source
M. acetivorans C2A	Wildtype *M. acetivorans**	J. G. Ferry
E. coli DH5α-λ*pir*	F^{-} *endA1 glnV44 thi-1 recA1 relA1 gyrA96 deoR nupG* φ80d*lacZ*ΔM15 Δ(*lacZYA-argF*)*U169 hsdR17*(r_K^{-} m_K^{+}) λ*pir*	W. W. Metcalf
pES1	Amp^R, Pur^R, R6K *ori*, C2A *ori*, P_{cdh}	K. R. Sowers
pES1(Pmat)	Amp^R, Pur^R, R6K *ori*, C2A *ori*, $P_{mcr\_ANME\text{-}1}$	This study
pES1-MAT*mcr*1	Amp^R, Pur^R, R6K *ori*, C2A *ori*, P_{cdh}::$mcr_{ANME\text{-}1}$	This study
pES1-MAT*mcr*2	Amp^R, Pur^R, R6K *ori*, C2A *ori*, $P_{mcr\_M.\ acetivorans}$::$mcr_{ANME\text{-}1}$	This study
pES1-MAT*mcr*3	Amp^R, Pur^R, R6K *ori*, C2A *ori*, $P_{mcr\_ANME\text{-}1}$::$mcr_{ANME\text{-}1}$	This study
pES1-MAT*mcr*1-*flag*	Amp^R, Pur^R, R6K *ori*, C2A *ori*, P_{cdh}::$mcr_{ANME\text{-}1}$-*flag*	This study
pES1-MAT*mcr*2-*flag*	Amp^R, Pur^R, R6K *ori*, C2A *ori*, $P_{mcr\_M.\ acetivorans}$::$mcr_{ANME\text{-}1}$-*flag*	This study
pES1-MAT*mcr*3-*flag*	Amp^R, Pur^R, R6K *ori*, C2A *ori*, $P_{mcr\_ANME\text{-}1}$::$mcr_{ANME\text{-}1}$-*flag*	This study

*In comparison to the reference genome of *M. acetivorans* (Genbank accession NC_003552.1), our wildtype *M. acetivorans* contains five amino acid substitutions, as revealed by genome sequencing: H313Q in MA2328 (hypothetical protein), A255T in MA1265 (DNA-directed RNA polymerase, beta subunit), A389T in MA0003 (sodium:proline symporter), F64L in MA2304 (phosphoserine aminotransferase), and W122R in MA2577 (hypothetical protein).

Supplementary Table 4. **Oligonucleotides used for cloning, sequencing, and verification of chromosomal and plasmid-based genes.** "f" indicates forward primer and "r" indicates reverse primer. All restriction enzyme sites used for cloning are underlined. Sequence for the FLAG tag is boxed. "*Phos*" indicates that the oligonucleotide is phosphorylated at the 5' end.

Primer name	Sequence (5'→3')
**Cloning of pES1-MAT*mcr*1 and sequencing of ANME-1 *mcrBGA* genes**	
veri-p-f	TAGTAGGTTGAGGCCGTTGAGC
pES1-f	CAATATGCCGCTTATCAGGATTAC
MATmcrB2-f	TATAGCCTGATAAAAGACAACG
MATmcrB3-f	ATCCATCCACCATTGGAAGAG
MATmcrB4-r	CAGGATGTCCATGTTGAGCTTG
pES1-r	CTTTGTTAGCAGCCGGATCTC
**Cloning of pES1-MAT*mcr*2**	
Pmcr-f2	GAATTCGGCTTCACGGTACCTTCTGCAGCACGGTATTATTAAACTTTCGTAT TAAGAGG
Pmcr-r2	TTGCCTTTGTCGTCATATAAATCTATTTCATCTGCCATTTTAATTTCCTCCTTA ATTTATTAAAATC
B6-r1	CAGTCATGCTAGCTTACAGTTTTGCACCGGGTTC
**Cloning of pES1-MAT*mcr*3**	
Pmat-f	GAATTCGGCTTCACGGTACCTTC
Pmat-r	TTGCCTTTGTCGTCATATAAATC
B6-r1	See above.
**Cloning of pES1(Pmat)**	
pES1(Matprom)-f	*Phos*-GCTAGCATGACTGGTGGACAGC
pES1(Matprom)-r	*Phos*-ATCTTCTTTTCCTCCTTTTACTATTACCC
**Cloning of pES1-MAT*mcr*1-*flag***	
pES1-f	See above.
B6-r-flag	AGTCATGCTAGCTTACTTGTCATCGTCGTCCTTGTAGTCCAGTTTTGCACCG GGTTCTC
**Cloning of pES1-MAT*mcr*2-*flag***	
Pmcr-f2	See above.
B6-r-flag	See above.
**Cloning and pES1-MAT*mcr*3-*flag***	
Pmat-f	See above.
B6-r-flag	See above.
**16S rDNA amplification and sequencing**	
ARCH109-F	AHDGCTCAGTAACACRT
ARCH934-R	GTGCTCCCCCGCCAATTCCT
27F	AGAGTTTGATCMTGGCTCAG
1492R	CGGTTACCTTGTTACGACTT
**Plasmid-based ANME-1 *mcrA* amplification**	
B4-f	ATACACTGGAAAAGGTCAGTA
pES1-r	See above.
**Chromosomal *mcrCD* amplification**	
mcrB-f	AACATCTGGCCTCATCGGTGAC
mcrG-r	CCCTGAGTTTCTCAAGTTTTCC

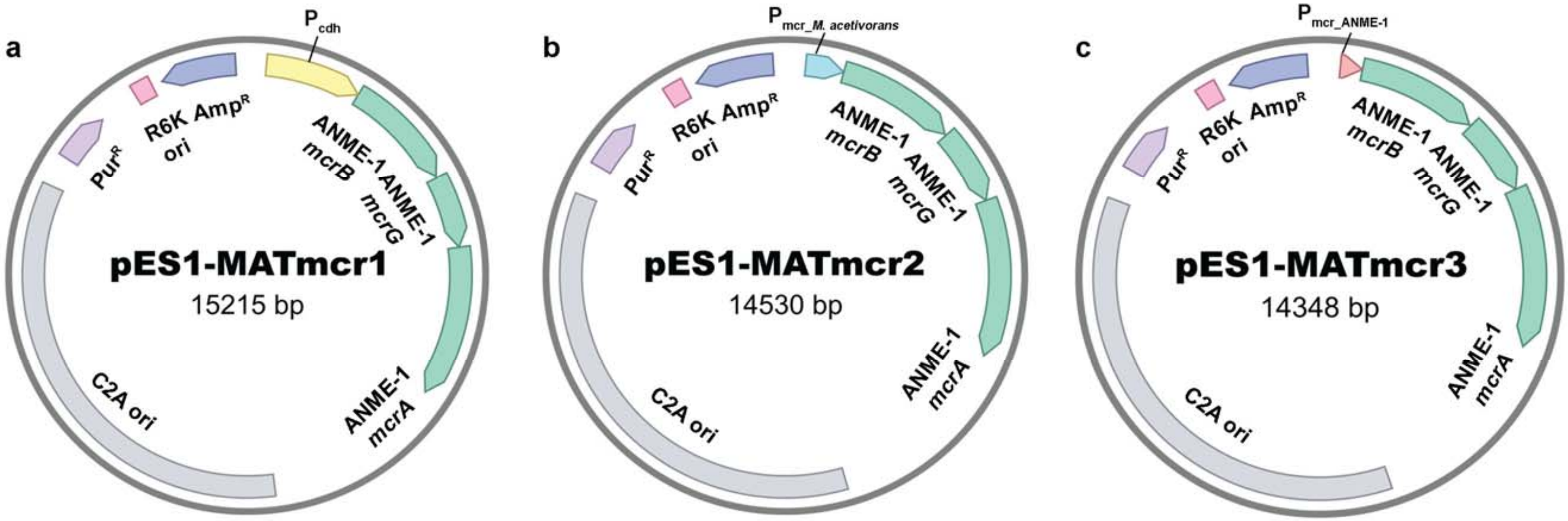

Supplementary Figure 1. Promoters used for expressing ANME-1 *mcrBGA*. Plasmid map of (a) pES1-MAT*mcr*1 with the CO dehydrogenase/acetyl-CoA synthase promoter (P_{cdh}) from *M. thermophila* for ANME-1 *mcrBGA*, (b) pES1-MAT*mcr*2 with the *mcr* promoter from *M. acetivorans* ($P_{mcr\_M.\ acetivorans}$) for ANME-1 *mcrBGA*, and (c) pES1-MAT*mcr*3 with the native *mcr* promoter from ANME-1 ($P_{mcr\_ANME\text{-}1}$) for ANME-1 *mcrBGA*. Similar plasmids were constructed with the sequence added for the FLAG tag to *mcrA* (see **Supplementary Table 4**).

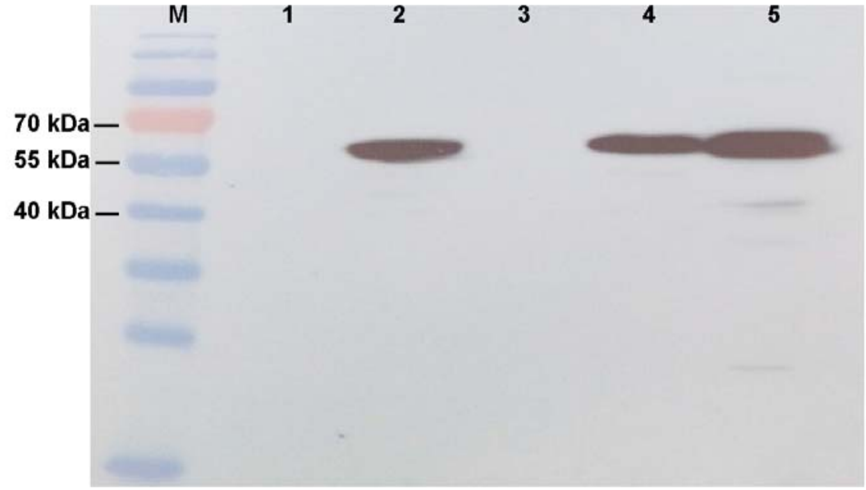

Supplementary Figure 2. Detection of ANME-1 McrA-FLAG in *M. acetivorans*/pES1-MAT*mcr*3 grown on methane after five days. Western blot shows ANME-1 McrA-FLAG (65 kDa) in *M. acetivorans*/pES1-MAT*mcr*1-*flag* (lane 2) and *M. acetivorans*/pES1-MAT*mcr*3-*flag* (lane 4) grown on methane and 0.1 mM $FeCl_3$ after five days (in high cell-density cultures). *E. coli* DH5α-λ*pir*/pES1-MAT*mcr*3 (lane 5) was used as the positive control. Lanes 1 and 3 correspond to methane-contacted *M. acetivorans* and *M. acetivorans*/pES1-MAT*mcr*2-*flag*, respectively.

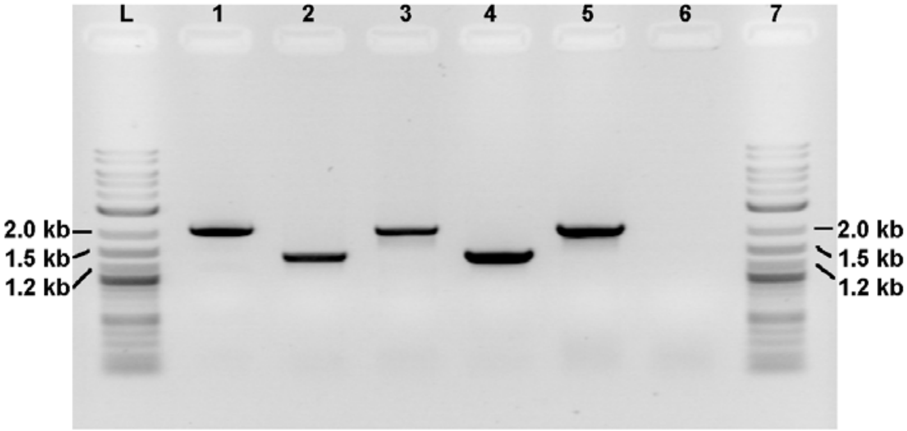

Supplementary Figure 3. Detection of ANME-1 *mcrA* after 30 days of growth on methane. PCR amplification of plasmid-based ANME-1 *mcrA* (2 kb, lanes 1, 3, and 5) and chromosomal-based *mcrCD* (1.3 kb, lanes 2, 4, and 6). DNA template for lanes 1 and 2: *M. acetivorans*/pES1-MAT*mcr*1 after 30 days (long-term) growth on methane and 0.1 mM $FeCl_3$. DNA template for lanes 3 and 4: freshly transformed *M. acetivorans*/pES1-MAT*mcr*1 growing on methanol. DNA template for lanes 5 and 6: *E. coli* DH5α-λ*pir*/pES1-MAT*mcr*1.

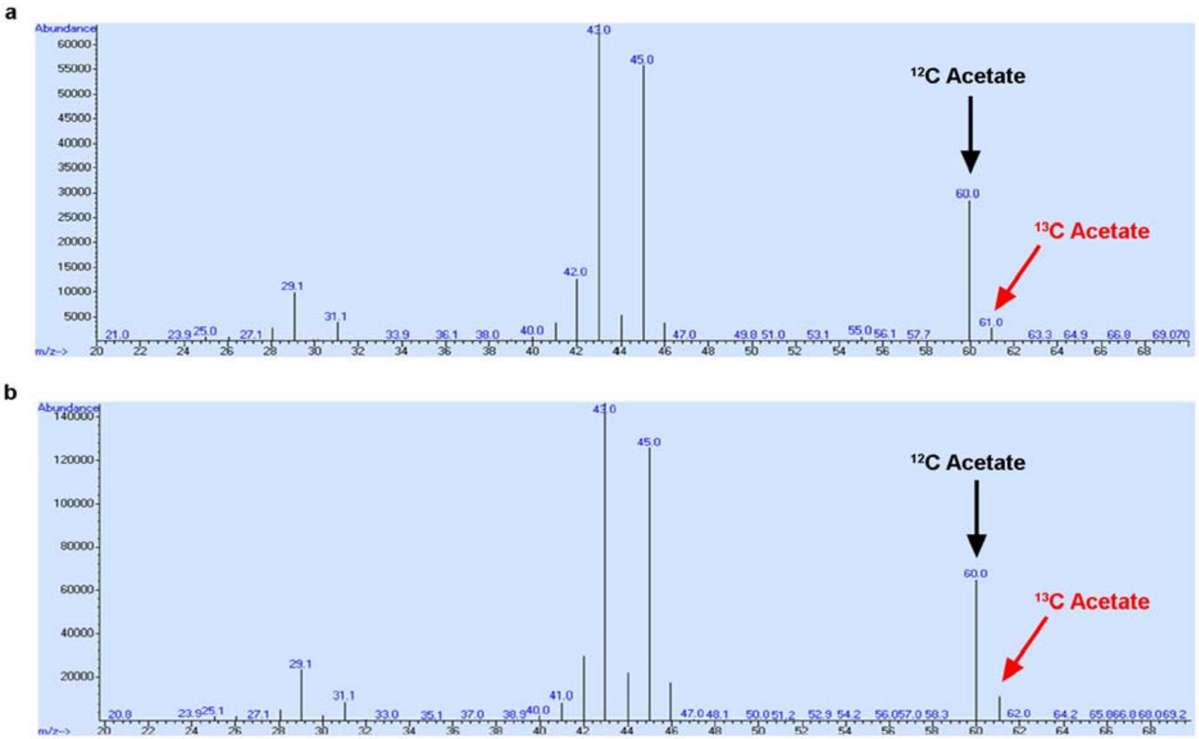

Supplementary Figure 4. Extracellular production of acetate from $H^{13}CO_3$ in ANME-1 Mcr-producing *M. acetivorans* via GC/MS. GC/MS spectra of culture supernatants used to identify acetate from $H^{13}CO_3$. Unlabeled acetate (^{12}C Acetate, black arrows) has a molecular mass of 60, whereas ^{13}C-labeled acetate (^{13}C Acetate, red arrows) has a molecular mass of 61. (a) *M. acetivorans* harboring empty plasmid pES1(Pmat) grown on ^{13}C-labeled bicarbonate for 10 days. (b) *M. acetivorans* harboring pES1-MAT*mcr*3 grown on ^{13}C-labeled bicarbonate for 10 days. Note that the y-axes differ for panels (a) and (b).

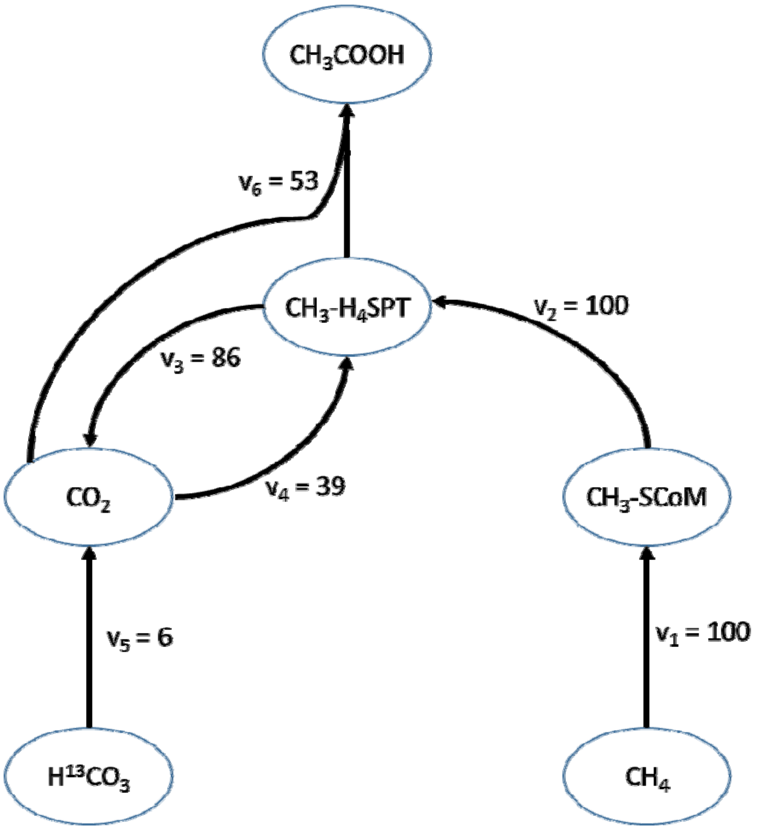

Supplementary Figure 5. Flux through the various reactions in the methanogenesis pathway of *M. acetivorans* estimated by ^{13}C-metabolic flux analysis using ^{13}C-labeled bicarbonate ($H^{13}CO_3$) as the input tracer. Methane uptake (v1) fixed at 100 mmol/gdw-h was used as the basis. The reduction of CO_2 in reaction v6 results in the incorporation of $H^{13}CO_3$ into the carbonyl carbon of acetate. The reverse flux through the methylotrophic pathway (v4) results in the incorporation of ^{13}C-bicarbonate into the methyl group of acetate, so both positions of acetate are labeled by $H^{13}CO_3$. Complete oxidation of methane to CO_2 via the methylotrophic pathway (v3) produces an unlabeled CO_2 pool resulting in a large unlabeled fraction of acetate.

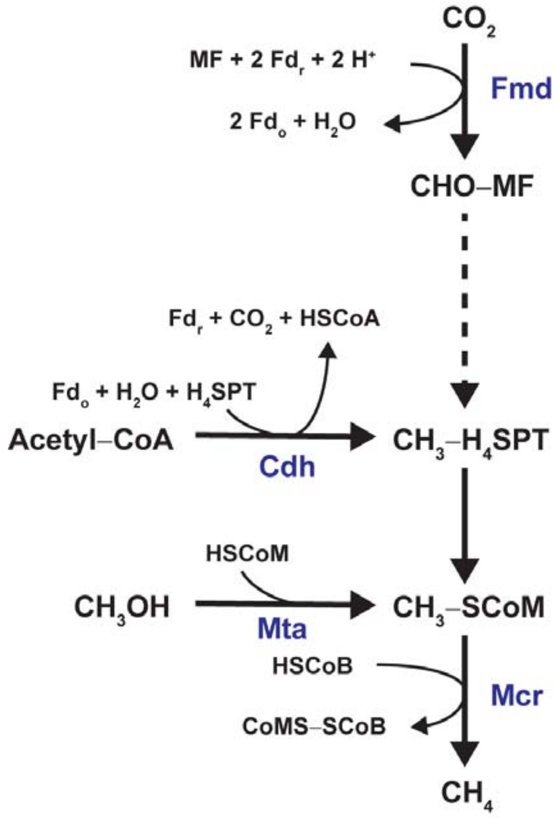

Supplementary Figure 6. Simplified methanogenesis pathway from CO_2 and CH_3OH of *M. acetivorans*. Enzymes in blue are multienzyme complexes, and one or two gene(s) encoding their subunit is repressed upon growth on methane (see **Supplementary Table 2** for details). Solid arrows denote reaction catalyzed by the enzymes. Dashed arrows denote shortened steps of catalytic transformations. Enzymes: Fmd, formyl-methanofuran dehydrogenase; Cdh, CO dehydrogenase/acetyl-CoA synthase; Mta, methanol-specific methyltransferase; and Mcr, methyl-coenzyme M reductase of *M. acetivorans* (not cloned ANME-1 Mcr). Metabolites: MF, methanofuran; Fd_r, reduced ferredoxin; Fd_o, oxidized ferredoxin; CHO-MF, formyl methanofuran; H_4SPT, 5,6,7,8-tetrahydrosarcinapterin; CH_3-H_4SPT, methyl-H_4SPT; CH_3-SCoM, methyl-coenzyme M; HSCoM, coenzyme M; HSCoB, coenzyme B; and CoMS-SCoB, heterodisulfide of coenzyme M and coenzyme B.
